# Targeting the Gut–Brain Axis: Protective Effects of NMN in Alleviating D-Galactose-Induced Cognitive Deficits

**DOI:** 10.3390/metabo16050314

**Published:** 2026-05-06

**Authors:** Zhenyang Zang, Feng Chen, Qiulian Tang, Wang Luo, Yuxian Lin, Jianxin Li, Yingcong Yu

**Affiliations:** 1 The Wenzhou Third Clinical Institute Affiliated to Wenzhou Medical University, Wenzhou 325035, China; 17276070722@163.com (Z.Z.); chenf0225@163.com (F.C.); 17320301799@163.com (Q.T.); luowang2025@163.com (W.L.); 2 Key Laboratory of Molecular Pharmacology and Drug Evaluation, Ministry of Education, School of Pharmacy, Yantai University, Yantai 264005, China; linyuxian0815@126.com; 3 School of Disaster and Emergency Medicine, Tianjin University, Tianjin 300072, China

**Keywords:** nicotinamide mononucleotide, aging, D-galactose, gut microbiota, oxidative stress

## Abstract

**Background**: To evaluate the neuroprotective effect of nicotinamide mononucleotide (NMN), an NAD^+^ precursor, in a D-galactose-induced aging mouse model. Chronic D-galactose administration is widely used to establish age-related cognitive impairment driven by oxidative stress. **Methods**: Mice received subcutaneous D-galactose for six weeks, concomitantly with oral NMN (300 or 500 mg/kg). Cognitive function was assessed using the Y-maze test and the Elevated Plus Maze test. Oxidative stress indicators, inflammatory cytokines, and Nrf2/HO-1 pathway components were measured by ELISA, Western blotting, and Immunohistochemistry. Gut microbiota composition was analyzed via 16S rRNA sequencing. **Results**: NMN supplementation improved spatial memory without affecting anxiety-related behavior. NMN enhanced the activities of antioxidant enzymes (SOD, GSH, CAT), reduced malondialdehyde and pro-inflammatory cytokine levels and decreased microglial activation in the hippocampus. Furthermore, NMN remodeled the gut microbiota by increasing butyrate-producing taxa (such as *Butyrivibrio_A* and *Clostridium_T*) and activated the Nrf2/HO-1 signaling pathway. **Conclusions**: NMN alleviates age-related cognitive decline in mice by reducing oxidative stress, suppressing neuroinflammation, and modulating the gut microbiota. Targeting the gut–brain axis and the Nrf2/HO-1 pathway may therefore represent a promising therapeutic strategy for age-related neurodegeneration.

## 1. Introduction

Aging is a complex and inevitable process of decline in physiological homeostasis that occurs as time passes [1]. Clinically, aging significantly heightens vulnerability to age-related neurodegenerative conditions, such as cognitive decline and extensive neuronal loss [2]. Under these circumstances, microglia, the immune cells in the central nervous system, play a dual role. Under physiological conditions, these myeloid cells maintain brain homeostasis by phagocytosing cellular debris and misfolded proteins [3]. However, accumulating evidence suggests that aging-associated microglial dysfunction promotes the accumulation of neurotoxic aggregates [4,5] and thereby accelerates the progression of brain aging and cognitive decline [6]. Specifically, age-related mitochondrial dynamic disorders impair microglial phagocytic and immunoregulatory capacity [7,8], while their sustained pro-inflammatory state further exacerbates synaptic loss and neuronal death during the aging process.

Brain aging arises from a complex interplay of genetic, molecular, and environmental factors. The gut microbiota has gradually been acknowledged as an important regulator of the age-associated neurodegenerative process [9,10]. The microbiota–gut–brain axis is a two-way communication system that affects CNS function and realizes gut-to-brain signal transmission [11]. Perturbations within this system—such as oxidative stress induced by prolonged D-gal exposure, immune senescence, metabolic dysregulation, and altered gut microbiota composition with reduced diversity [12]—collectively contribute to dysbiosis. Subsequent shifts in microbial community structure facilitate pathogenic colonization and the accumulation of neurotoxic metabolites [13]. Gut microbiota dysbiosis is increasingly recognized as an important risk factor for CNS pathophysiology in the aging brain [14,15,16]. Microbially-mediated disruptions initiate systemic inflammatory cascades that compromise blood–brain barrier integrity, thereby accelerating neuroinflammatory signaling and neuronal degeneration [17,18]. A growing number of studies have shown that changes in the gut microbiota can induce cognitive decline in older adults; in addition, dysregulated inflammatory factors and neuroactive microbial metabolites are also involved in age-related cognitive decline [19,20,21,22,23]. Notably, while commensal gut microorganisms in a homeostatic state continuously produce anti-inflammatory [24] and neuroprotective metabolites [25], age-related dysbiosis shifts this balance toward a pro-inflammatory milieu that favors neurodegeneration. Comparative metagenomic analyses of fecal microbiomes from aged individuals with cognitive impairment versus age-matched cognitively intact counterparts have consistently demonstrated marked reductions in microbial diversity, alongside selective overgrowth of pathobionts and depletion of beneficial taxa in age-associated dysbiosis [26]. These collective observations have generated interest in gut microbiota modulation as a viable therapeutic strategy for mitigating brain aging. Oral administration of nicotinamide mononucleotide (NMN) has shown specific applications’ value for cognitive function protection intervention [27,28,29,30]. As the primary precursor of NAD^+^ and a well-recognized anti-aging nutraceutical, NMN has drawn the attention of both scientists and the healthcare and wellness industry for consumers [31]. It has been documented to exert multiple pharmacological effects on metabolism, including but not limited to regulating endocrine function, enhancing insulin secretion to ameliorate obesity and diabetes [32]; improving sleep quality [33]; promoting DNA repair mechanisms [34]; and restoring liver metabolic homeostasis [35]. Furthermore, NMN has been shown to improve intestinal barrier function, thereby further supporting its potential application in the fields of aging and neurodegeneration [36].

Despite accumulating evidence supporting the neuroprotective effects of NMN, whether it can counteract age-related cognitive decline through regulation of the gut microbiota has not been explored either. The gut–brain axis has become a key regulatory factor for CNS Function, and interventions that restore microbial homeostasis may offer unique therapeutic advantages. At present, using a D-galactose-induced aging mouse model in this study aims to systematically examine the effects of NMN on learning and memory impairments. We conjecture that NMN alleviates cognitive impairment by suppressing oxidative stress and neuroinflammation via the Nrf2/HO-1 pathway activation and modulating the gut microbiota community. Our findings demonstrate that oral NMN administration improves cognitive function in a dose-dependent manner, reduces microglial activation, enriches butyrate-producing gut bacteria, and upregulates Nrf2/HO-1 signaling. These results position NMN as a multimodal intervention targeting both central and peripheral mechanisms of brain aging.

## 2. Materials and Methods

### 2.1. Animal Models and Supplementation Protocols

**Figure 1:**
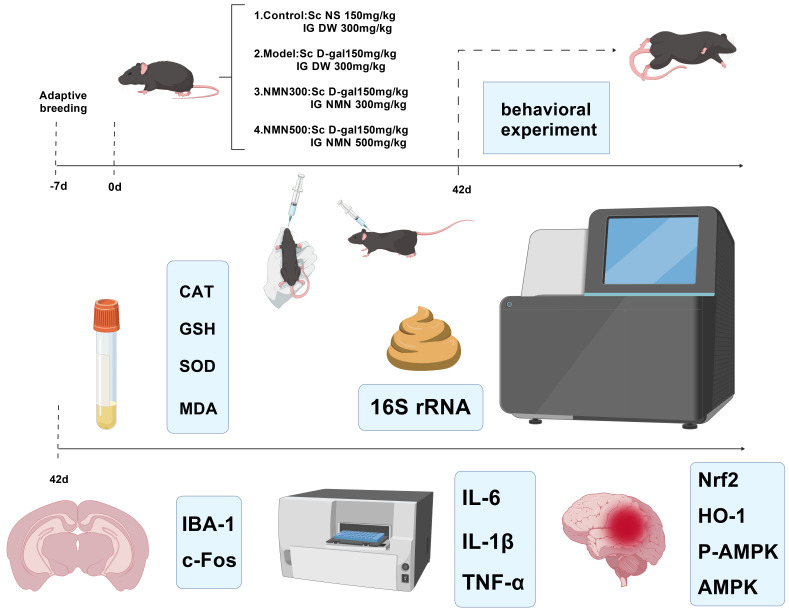
Experimental flow chart. Abbreviations: Sc, subcutaneous injection; NS, normal saline; IG, intragastric administration; DW, distilled water.

Four-month-old male C57BL/6J mice were supplied by Beijing Viton Life Science Experimental Animal Technology Co., Ltd. (Beijing, China). All animal experiments were approved by the Institutional Animal Care and Use Committee of Oujiang Laboratory (approval No. OJLAB24122001). Mice were housed individually in a standard environment (22 ± 2 °C, 50 ±10%rh, 12 h light-dark cycle), with free access to standard laboratory mouse chow and drinking water. After one week of adaptation, animals were randomly divided into four groups (*n* = 7 each): control (vehicle), model (D-galactose), NMN 300 mg/kg (Int1) [37], and NMN 500 mg/kg (Int2) [38]. A post hoc power analysis based on the primary outcome (spontaneous alternation rate in Y-maze) indicated that with *n* = 7 per group, the achieved statistical power was 0.87 (α = 0.05, observed effect size Cohen’s d = 1.59), which exceeds the conventionally acceptable level of 0.80. This sample size is also consistent with previous similar mouse studies of aging and cognitive function. The control group of mice was injected subcutaneously with normal saline, and the aging model groups were given D-galactose (D-gal; Muke Biology Co., Ltd. (Wenzhou, China)) by subcutaneous injection at a dosage of 150 mg/kg/day for six weeks to induce age-related changes. Parallel to D-galactose supplementation, the control and model groups were given a vehicle (saline) by oral gavage, while the supplementation groups received oral NMN at a daily dosage of 300 mg/kg or 500 mg/kg. Following the 42-day intervention, behavioral tests (Y-maze and Elevated Plus Maze) were performed; animals were then euthanized, and biological specimens were harvested for further analysis (Figure 1). All the procedures were strictly followed by the institution’s regulations to reduce the animals’ suffering and maximize experimental efficiency.

### 2.2. Behavioral Test

Following 42 days of D-galactose administration, spatial working memory was tested by the Y-maze test, as described previously. The Y-maze equipment was made up of three equal arms (40 cm × 10 cm × 15 cm) and arranged at 120-degree angles in an equilateral triangular configuration. Before each test, we cleaned the device thoroughly with 75% ethanol and dried it in the air to remove any lingering odor from other people that may influence foraging behavior. Individual mice were placed at the center of the Y-maze and allowed to move freely for eight minutes across the three arms (A, B, and C). An entry was recorded if any four paws entered an arm simultaneously. The number of sequential and total Entries was recorded, and then the spontaneous alternation rate was calculated based on this: [(spontaneous alternations)/((total entries)−2)] × 100%.

The Elevated Plus Maze (EPM) was used to examine the anxiety-like behavior. The device was composed of two open arms and two closed arms (both were 50 cm × 10 cm, and there were transparent walls of 40 cm high on the latter), with a central platform that was raised by 50 cm and had dimensions of 5 cm × 5 cm. The lights were dimmed, and the mouse was allowed to explore the open arms. Each animal was placed in the central platform, facing one of the open arms, and free exploration was allowed for 5 min. Between trials, the apparatus was wiped with 75% ethanol and air-dried before the next arm-entry test.

### 2.3. Western Blot

Mice were euthanized under anesthesia, and the liver and hippocampus tissues were rapidly dissected, snap-frozen in liquid nitrogen, and stored at −80 °C until further processing. Hippocampal tissue homogenates were prepared using an RIPA lysis buffer containing protease and phosphatase inhibitors to prevent protein breakdown. Clarify after centrifugation at 4 °C, determine the protein concentration of lysates using BCA reagents to obtain an equal amount per lane. Proteins were denatured by adding DTT and SDS before separating them using SDS-PAGE. Electrophoresis conditions: At 70 V for 20 min (stacking) and then at 110 V for 60 min (separating). Wet transfer of PVDF membranes concomitantly with blocking with 5% skim milk in TBST. Primary antibodies against IL-6, IL-1, TNF-, IBA-1, Nrf2, HO-1, P-AMPK, AMPK, and β-actin were added overnight at 4 °C (dilution: 1:1000–1:10,000; supplier: Proteintech (Wuhan, China) or Abcam (Cambridge, UK)). TBST was washed after that, then HRP-conjugated secondary antibodies were added (Cell Signaling Technology, Danvers, MA, USA) for 2 h at room temperature. Bands were developed using ECL, and band densities were quantified via ImageJ (version 2.2, NIH). Protein expression was normalized to β-actin and expressed relative to controls.

### 2.4. IHC Immunohistochemistry

Coronal brain sections at equivalent anatomical levels in all animals were processed in sequence with 3 sets of PBS washes (5 min). Then the sections were washed with PBST (0.3% Triton X-100 in PBS), and the rabbit anti-c-Fos primary antibody (Bioss Pathology Company, Wuhan, China; 1:2000 dilution) was added and incubated overnight at 4 °C with orbital shaking. After three more washes in PBS (5 min each), the sections were incubated with donkey anti-rabbit IgG secondary antibody (Bioss Pathology Company, Wuhan, China) at a 1:2000 dilution for 2 h at room temperature. After the final PBS washing series (three times, 5 min each), sections were mounted on positively charged glass slides according to coronal A-P orientation, air dried, and coverslipped. Neuronal activation was assessed by quantifying c-Fos-positive cells. Images were captured using an Olympus VS120 microscope (Olympus Corporation, Tokyo, Japan). Scale bars were added to all IHC images using the microscope software (Olympus VS-ASW, Olympus Corporation, Tokyo, Japan).

The immunohistochemical staining color intensity was quantitatively evaluated by histostain score (H score), a common semiquantitative way to assess the percentage and extent of labeled immune cells. The H-score was derived as Σ (i × Pi), where i represents staining intensity (graded 1–3), and Pi denotes the percentage of cells positive at each intensity level. Specifically, the final score was calculated as: (1 × percentage of cells with intensity 1) + (2 × percentage of cells with intensity 2) + (3 × percentage of cells with intensity 3).

The number of c-Fos-positive cells was quantified as “Num Positive per mm^2^”, which represents the count of positively stained nuclei per square millimeter of hippocampal tissue section.

### 2.5. IF Immunofluorescence

Following fixation in 4% paraformaldehyde, brain tissues were paraffin-embedded and sectioned at 5 μm. Putting the slides in the oven to bake them, passing them through a series of ethanol solutions for dehydration, and then treating them with sodium citrate buffer under heat for antigen retrieval. Peroxidase activity was quenched with 3% H_2_O_2_ in PBS, and non-specific binding was minimized using 10% goat serum (40 min). Anti-IBA-1 polyclonal antibody (1:500; Proteintech, Wuhan, China) was added overnight at 4 °C in a humidified box to assess microglial activation. After returning to room temperature for 45 min and three PBS washes, the sections were then incubated with an Alexa Fluor 488-conjugated donkey anti-rabbit secondary antibody (green fluorescence; 1:2000; Bioss, Wuhan, China) for one hour in darkness. Nuclear counterstaining was achieved with DAPI-containing mounting medium (10 min), concomitantly with three PBS washes and coverslipping. IBA-1 immunoreactivity was examined under fluorescence microscopy, and the area of IBA-1 positivity was quantified using ImageJ. All immunofluorescence images were acquired using an Olympus VS120 virtual slide scanning microscope (Olympus Corporation, Tokyo, Japan). Scale bars were added to all IF images, as indicated in the figure legends.

IBA-1 immunoreactivity was quantified as “Positive, %”, defined as the percentage of IBA-1-positive area relative to the total examined area in the hippocampal region.

### 2.6. ELISA Enzyme-Linked Immunosorbent Assay

Blood collected in non-anticoagulant tubes was allowed to clot (1 h at room temperature or overnight at 2–8 °C) before being centrifuged at 1000× *g* for 20 min at 2–8 °C to obtain serum; it was then divided into portions and kept. The hippocampal tissue was weighed, added to an equal volume of PBS (pH 7.4) solution, frozen quickly with liquid nitrogen, and stored at −80 °C. After thawing, the samples were placed on ice and homogenized in PBS; after centrifugation at 2000–3000× *g* for 20 min, the supernatant was collected. Oxidative stress indicators (GSH, SOD, CAT, MDA) and inflammatory cytokines were detected by an ELISA kit (Nanjing Jiancheng Bioengineering Research Institute, Nanjing, China). Using a microplate reader to find the value of each substance according to the standard curve.

### 2.7. DNA Extraction and 16S rRNA Gene Sequencing Analysis

Fresh fecal samples were collected from each mouse, immediately snap-frozen in liquid nitrogen, and stored at −80 °C until processing. Total genomic DNA was extracted using the E.Z.N.A.^®^ Stool DNA Kit (Omega Bio-tek, Norcross, GA, USA) according to the manufacturer’s instructions. DNA concentration and purity were assessed using a NanoDrop 2000 spectrophotometer (Thermo Scientific, Waltham, MA, USA), and integrity was verified by 1% agarose gel electrophoresis.

The V3–V4 hypervariable region of the bacterial 16S rRNA gene was amplified using the primer pair 338F (5′-ACTCCTACGGGAGGCAGCA-3′) and 806R (5′-GGACTACHVGGGTWTCTAAT-3′). PCR amplification was performed in a 20 μL reaction volume containing 10 ng of template DNA, 0.8 μL of each primer (10 μM), and 10 μL of 2× KAPA HiFi HotStart ReadyMix (Kapa Biosystems, Wilmington, MA, USA). Thermal cycling conditions were as follows: initial denaturation at 95 °C for 3 min; 25 cycles of 95 °C for 30 s, 55 °C for 30 s, and 72 °C for 30 s; and a final extension at 72 °C for 5 min. PCR products were purified using an AxyPrep DNA Gel Extraction Kit (Axygen Biosciences, Union City, CA, USA) and quantified with a Qubit 4.0 fluorometer (Thermo Fisher Scientific, Waltham, MA, USA).

Sequencing libraries were prepared using the TruSeq Nano DNA Library Prep Kit (Illumina, San Diego, CA, USA) and sequenced on an Illumina NovaSeq 6000 platform (Illumina, San Diego, CA, USA) with 2 × 250 bp paired-end chemistry at Muke Biotechnology Co., Ltd. (Hangzhou, China).

Raw sequencing reads were processed using QIIME2 (version 2023.2). Primer sequences were removed with the cutadapt plugin, and reads were quality-filtered, denoised, merged, and chimera-checked using the DADA2 pipeline to generate amplicon sequence variants (ASVs). Taxonomic assignment was performed using a pre-trained Naïve Bayes classifier based on the SILVA 138 reference database with a confidence threshold of 0.7.

For downstream analysis, ASV count data were normalized by relative abundance transformation for visualization. Differential abundance analysis was conducted using DESeq2 with false discovery rate (FDR) correction. Alpha diversity was assessed using Shannon and Chao1 indices, and group differences were evaluated by the Kruskal–Wallis test. Beta diversity was calculated using Bray–Curtis and unweighted UniFrac distances and visualized by principal coordinate analysis (PCoA). Statistical significance of community composition differences was determined by permutational multivariate analysis of variance (PERMANOVA) with 999 permutations. Linear discriminant analysis effect size (LEfSe) was performed to identify differentially abundant taxa, with a logarithmic LDA score threshold of 2.0 and FDR-adjusted * *p* < 0.05. Multiple-testing correction was applied using the Benjamini–Hochberg method throughout.

### 2.8. Statistical Analysis

The data are presented as the mean ± standard deviation (SD). Normality of the data was assessed using the Shapiro–Wilk test for each group, which confirmed that the data did not deviate significantly from a normal distribution (*p* > 0.05 for all groups). Between-group differences were then analyzed using one-way analysis of variance (ANOVA) followed by Tukey’s Honestly Significant Difference (HSD) post hoc test for multiple comparisons. The significance level was set at * *p* < 0.05.

## 3. Results

### 3.1. Body Weight, Food Intake and NAD^+^ Levels

**Figure 2:**
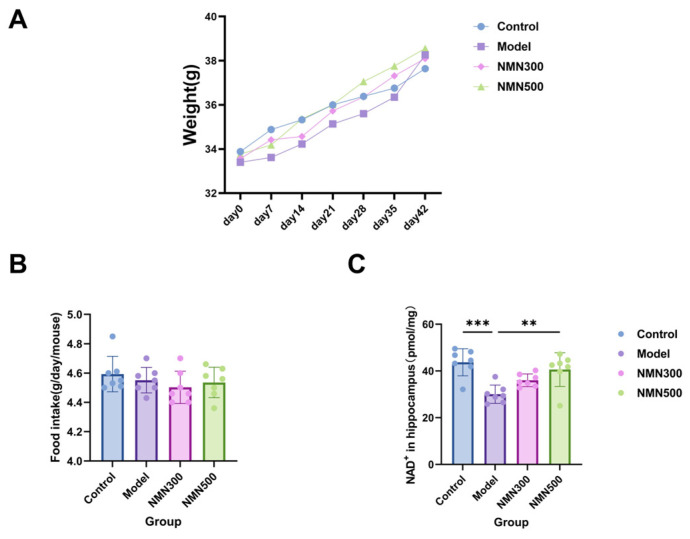
NMN administration does not affect body weight but restores NAD^+^ levels. (**A**) Body weight curves of mice in the control, model, NMN 300 mg/kg, and NMN 500 mg/kg groups during the 6-week treatment period; (**B**) food intake of each mouse daily in the control group, model group, NMN 300 mg/kg group and NMN 500 mg/kg group during the 6-week treatment period; (**C**) effect of NMN on the level of NAD^+^ in the brain tissue of D-gal-induced aging mice was determined by ELISA; ** *p* < 0.01, *** *p* < 0.001; *n* = 7; data were analyzed by one-way ANOVA, followed by Tukey’s HSD post hoc test for multiple comparisons. Data are expressed as means ± standard deviations.

Throughout the 6-week intervention, we monitored general physiological parameters to exclude confounding factors. As shown in Figure 2A, no significant differences were observed in initial body weight, final body weight, or body weight gain among the control, model, NMN 300 mg/kg and NMN 500 mg/kg groups (one-way ANOVA, *p* > 0.05), indicating that NMN did not alter general nutritional status.

Importantly, we further assessed the metabolic status by measuring NAD^+^ levels. As shown in Figure 2B, the model group showed a significant decline in NAD^+^ concentration compared to the control group, suggesting a metabolic impairment in the disease state. Notably, NMN supplementation (500 mg/kg) significantly restored NAD^+^ levels in a dose-dependent manner. These data demonstrate that although NMN did not affect body weight, it effectively ameliorated the metabolic deficit by replenishing the NAD^+^ pool.

### 3.2. NMN Attenuates Behavioral Deficits in D-Galactose-Induced Aging Mice

**Figure 3:**
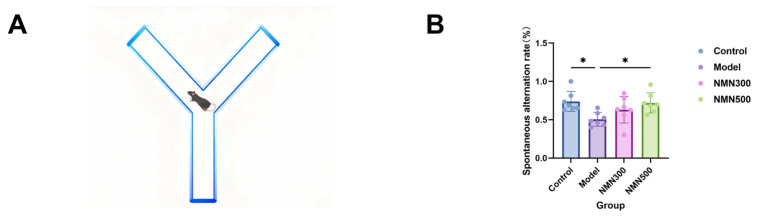
(**A**) Schematic diagram of the Y-maze device; (**B**) shows that NMN can increase the spontaneous alternation rate in D-gal-induced aging mice; * *p* < 0.05; *n* = 7; data were analyzed by one-way ANOVA, followed by Tukey’s HSD post hoc test for multiple comparisons. Data are expressed as means ± standard deviations.

To determine whether NMN can improve cognitive impairment in older mice caused by D-galactose, we assessed spatial working memory using the Y-maze test (Figure 3A). spatial working memory was tested via the Y-maze test (Figure 3A). As shown in Figure 3B, the spontaneous alternation rate was significantly lower in the model group than in the control group, and NMN supplementation at 500 mg/kg restored it to the control level (*p* < 0.05). These results show that D-galactose administration has impaired spatial memory, to some extent, which is reduced by NMN.

**Figure 4:**
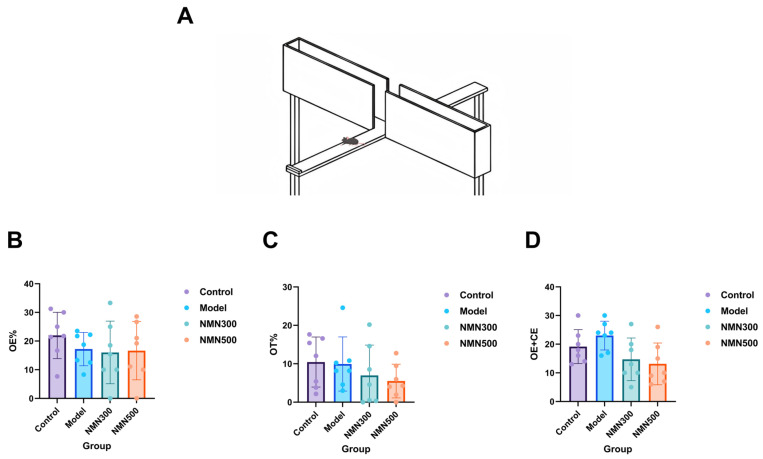
(**A**) Schematic diagram of the Elevated Plus Maze; (**B**) Evaluation of anxiety levels indicating that NMN treatment did not affect the behavior of D-galactose-induced aging mice in the Elevated Plus Maze.Open arm entry (OE): the number of times an animal enters any open arm, and 80% of the body has entered the arm for it to be considered. OE% = OE/(OE + CE) × 100%; (**C**) Open-arm activity (OT): the time when 80% of the body was in the open arm. OT% = time spent in the open arm/(time spent in the open arm + time spent in the closed arm) × 100%; (**D**) OE + CE (closed arm entries): the number of times an animal enters either closed arm, with 80 percent of its body inside the arm considered as entering; *n* = 7; data were analyzed by one-way ANOVA, followed by Tukey’s HSD post hoc test for multiple comparisons. Data are expressed as means ± standard deviations.

The Elevated Plus Maze test was used to examine anxiety-related behavior (Figure 4A). As shown in Figure 4B–D, none of the parameters—including open-arm entry percentage (OE%), open-arm time percentage (OT%), and total entries (OE + CE)—differed significantly between groups, suggesting that anxiety levels remained unaffected by the supplementations. Overall, these results indicate that NMN specifically enhances cognitive function without inducing significant anxiety in the D-galactose aging model.

### 3.3. NMN Enhances Hippocampal Neuronal Activation in Cognitive Impairment Mice

**Figure 5:**
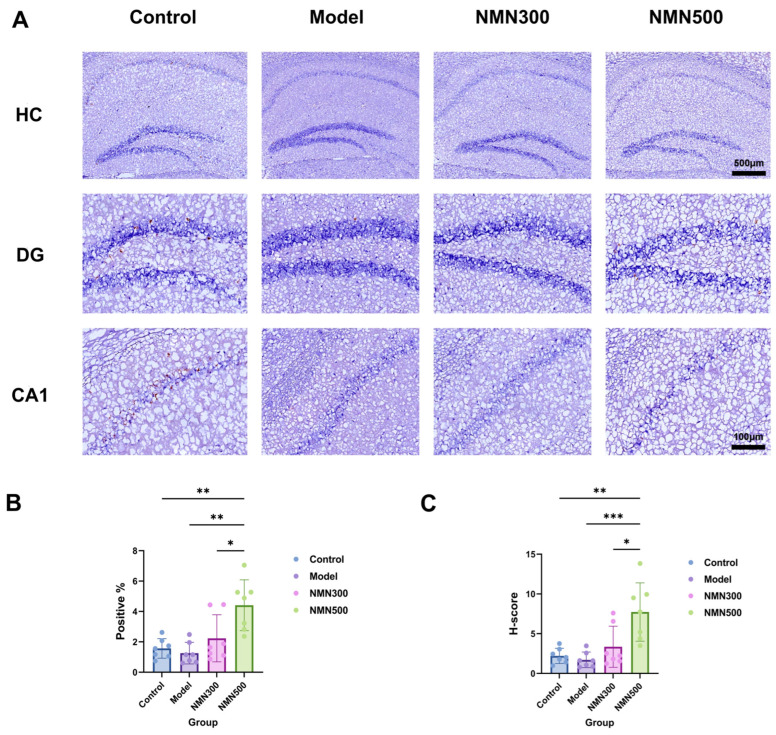
NMN raises c-Fos levels in the brains of D-gal-induced aging mice. (**A**) Immunohistochemical staining of c-Fos in the hippocampus of brain tissue. (**B**) Quantification of cells expressing c-Fos expressed as Num Positive per mm^2^ (number of positive cells per square millimeter). (**C**) c-Fos immunohistochemical score of brain tissue. * *p* < 0.05, ** *p* < 0.01, *** *p* < 0.001; *n* = 7; data were analyzed by one-way ANOVA, followed by Tukey’s HSD post hoc test for multiple comparisons. Data are expressed as means ± standard deviations.

Reduced neuronal activity is a key functional feature of progressive cognitive deterioration. The immediate early gene c-Fos serves as a reliable marker of neuronal activity. Upon neuronal stimulation, c-Fos transcription is rapidly upregulated, leading to the accumulation of Fos protein in the nuclei of activated neurons. As such, c-Fos immunoreactivity allows for evaluation of neuronal excitation, synaptic transmission efficacy and neuropsychiatric disease pathogenesis. Comparative immunohistochemical analysis of c-Fos distribution in the control, model, and NMN 500 mg/kg groups revealed marked differences (Figure 5A), indicating that D-galactose administration impaired neuronal activity. In hippocampal tissues, NMN supplementation led to marked increases in both the c-Fos immunoreactive area and the immunohistochemical H-score (Figure 5B,C), indicating alleviation of neuronal impairment.

As the brain’s resident macrophages and primary neuroimmune effectors, microglia maintain central nervous system (CNS) homeostasis under physiological conditions through metabolic regulation and facilitation of synaptic plasticity, learning, and memory consolidation. However, oxidative stress-driven microglial activation initiates neuroinflammatory cascades that are strongly involved in the pathophysiology of AD (Alzheimer’s disease), depression, dementia, and cognitive impairment. Oxidative stress-induced elevation of reactive oxygen species (ROS), including superoxide anions and hydroxyl radicals, can directly oxidize microglial proteins, lipids, and DNA, disrupting intracellular redox balance. This promotes phenotypic switching from resting to activated microglia by producing inflammatory factors (IL-6, TNF-α, IL-1β) as well as neurotoxic molecules. To determine the regulatory effect of NMN on this pathway, immunofluorescence staining technology was used to examine microglial activity status in the hippocampus of D-galactose-treated mice, and ELISA and Western blot analyses were performed to detect IL-6, TNF-α, and IL-1β contents in hippocampal tissue.

**Figure 6:**
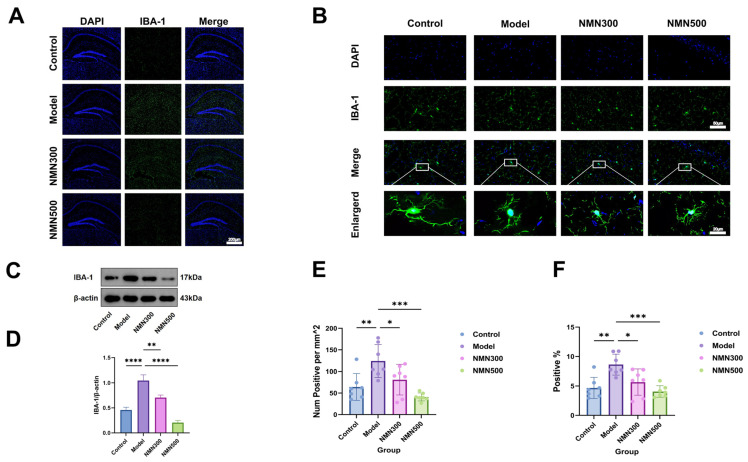
NMN can regulate the inflammation in the brain of D-gal-induced aging mice and alleviate the over-expression of microglia in the brain. (**A**) Schematic diagram of immunofluorescence staining of microglia in the hippocampal region of brain tissue. (**B**) representative immunofluorescent staining images of IBA-1 (green) with DAPI (blue). (**C**) Semi-quantitative analysis of IBA-1 expression shown as Positive, % (percentage of IBA-1-positive area relative to total examined area). (**D**) positive results of immunofluorescence of IBA-1 per mm^2^ in brain tissue; (**E**) positive results of immunofluorescence of IBA-1 in brain tissue. * *p* < 0.05, ** *p* < 0.01, *** *p* < 0.001, **** *p* < 0.0001; *n* = 7; Data were analyzed by one-way ANOVA, followed by Tukey’s HSD post-hoc test for multiple comparisons. Data are expressed as means ± standard deviations.

As illustrated in Figure 6, NMN supplementation effectively attenuated microglial activation and neuroinflammation in the D-galactose aging model. Quantification of IBA-1 immunofluorescence showed a significant increase in microglial activation in the Model group compared with the Control group (*p* < 0.01). NMN supplementation at 500 mg/kg significantly reduced IBA-1 expression compared with the Model group (*p* < 0.01). A significant increase in the density of activated microglia was observed in the Model group compared with the Control group (*p* < 0.01), and NMN at 500 mg/kg could restore it to a normal level (*p* < 0.01). The difference in IBA-1 expression between the Control and NMN 500 mg/kg groups was not statistically significant (*p* > 0.05), indicating that NMN supplementation restored microglial activation to baseline levels without inducing excessive suppression. Morphological characterization showed that the Control group microglia were in a state of rest, with small soma, elaborated ramified process and slender cellular architecture; In the Model group, microglia activated extensively and polarized, exhibiting an amoeboid form with a significant increase in cell density; NMN 500 mg/kg supplementation restored microglia morphology to the resting, ramified type consistent with decreased neuroinflammation.

**Figure 7:**
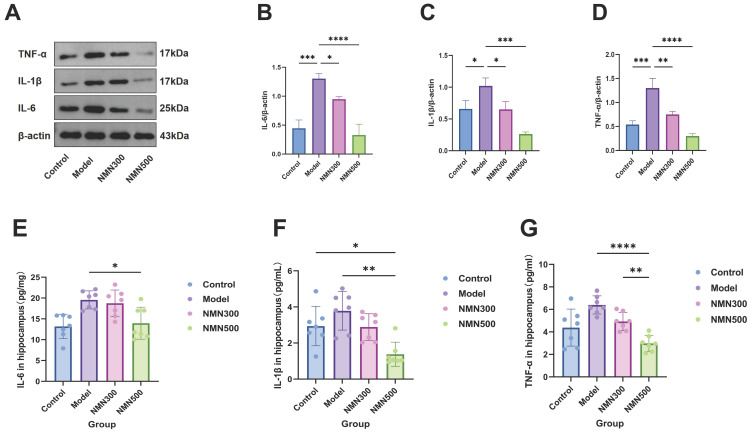
NMN can reduce the brain inflammation caused by aging in D-gal-induced-aging mice. (**A**) TNF-α, IL-1β and IL-6 expression in the brain was detected by Western blot. (**B**) Semi-quantitative analysis of IL-6 grey value. (**C**) Semi-quantitative analysis of IL-1β grey value. (**D**) Semi-quantitative analysis of TNF-α grey value. (**E**) Effect of NMN on the level of IL-6 in the brain tissue of D-gal-induced aging mice was determined by ELISA. (**F**) Effect of NMN on the level of IL-1β in the brain tissue of D-gal-induced aging mice was determined by ELISA. (**G**) Effect of NMN on the level of TNF-α in the brain tissue of D-gal-induced aging mice was determined by ELISA. * *p* < 0.05, ** *p* < 0.01, *** *p* < 0.001, **** *p* < 0.0001; *n* = 7; data were analyzed by one-way ANOVA, followed by Tukey’s HSD post hoc test for multiple comparisons. The data are expressed as the mean ± standard deviation.

As shown in Figure 7, the hippocampal expression of IL-6, TNF-α and IL-1β was significantly higher in the Model group than in the Control group by Western blot and ELISA. Administration of NMN at 300 or 500 mg/kg reversed this increase in a dose-dependent manner, and the high-dose group was more inhibited. Collectively, these results indicate that NMN mitigates neuroinflammation in the aging brain by downregulating these pro-inflammatory cytokines.

Western blotting and ELISA showed a decrease in the protein content of IL-6, TNF-α, and IL-1β after NMN supplementation. Notably, in the NMN 500 mg/kg group, the levels of these cytokines were slightly lower than those in the control group, although the difference did not always reach statistical significance. Collectively, these results strongly support the view that NMN has significant anti-neuroinflammatory effects in the hippocampus of D-gal-induced aged mice.

### 3.4. NMN Is Associated with the Composition of Gut Microbiota in Mice with Cognitive Impairment

**Figure 8:**
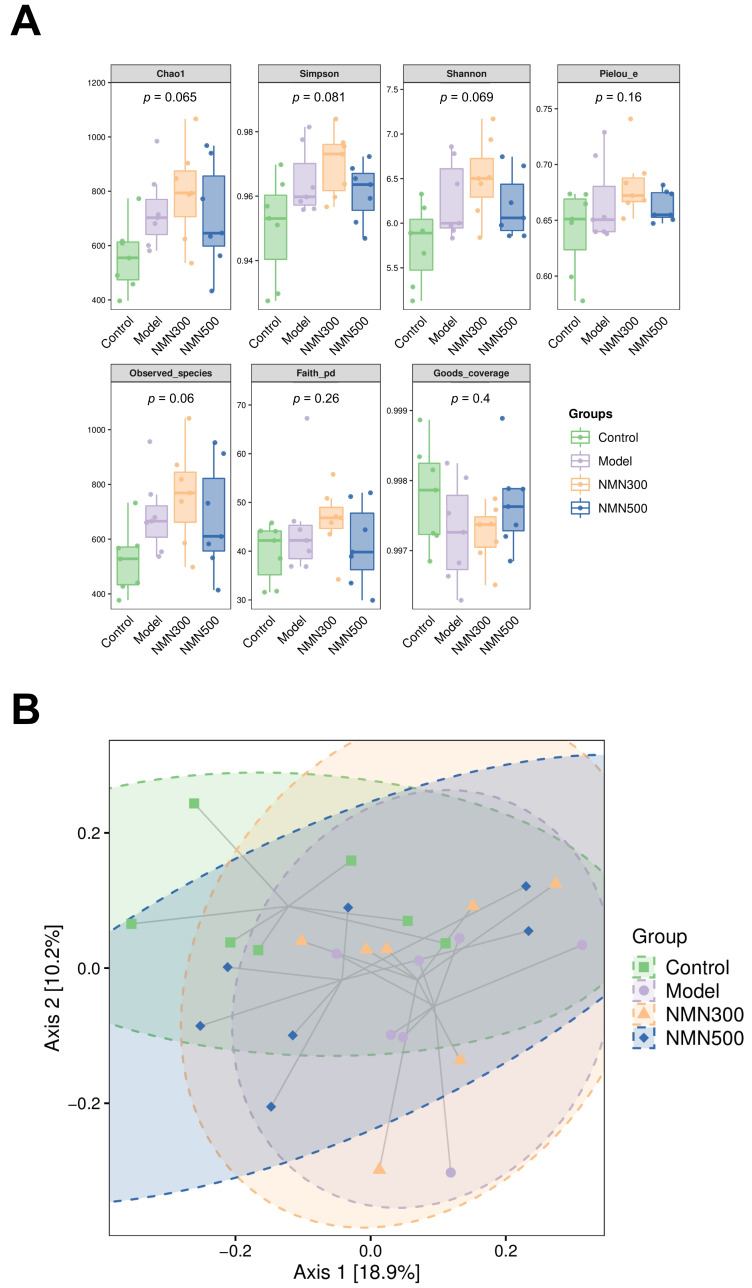
NMN can adjust the gut microbiota structure of D-gal-induced aging mice. (**A**) Alpha diversity indices (Chao1, Simpson, Shannon, Pielou_e, Observed_species, Faith_pd, and Goods_coverage) across the control, model, NMN 300 mg/kg, and NMN 500 mg/kg groups; (**B**) principal coordinate analysis (PCoA) of beta diversity based on Bray–Curtis dissimilarity. Each point represents an individual sample, and ellipses indicate 95% confidence regions for each group. Percentages on axes denote the proportion of variance explained by the corresponding principal coordinate; *n* = 7. For panel A, group differences were evaluated by the Kruskal–Wallis test. For panel B, significance of community composition differences was determined by PERMANOVA with 999 permutations. Data are expressed as means ± standard deviations.

Gut microbiota architecture among the four experimental groups was characterized by 16S rRNA high-throughput sequencing. Alpha diversity was evaluated using multiple indices (Chao1, Simpson, Shannon, Pielou_e, Observed_species, Faith_pd, and Goods_coverage; Figure 8A). Although no statistically significant differences were detected among groups by the Kruskal–Wallis test (*p* > 0.05 for all indices), a consistent trend toward higher community richness and evenness was observed in the NMN-supplemented groups compared with the model group.

Beta diversity was visualized by principal coordinate analysis (PCoA) based on Bray-Curtis dissimilarity (Figure 8B). The NMN 500 mg/kg group formed a distinct cluster that separated clearly from the model group along the first two principal coordinates (Axis 1: 18.9%, Axis 2: 10.2%), indicating a substantial NMN-induced shift in overall community composition. PERMANOVA confirmed that the differences in microbial community structure among groups were statistically significant (pseudo-F = 1.154, *p* = 0.03).

**Figure 9:**
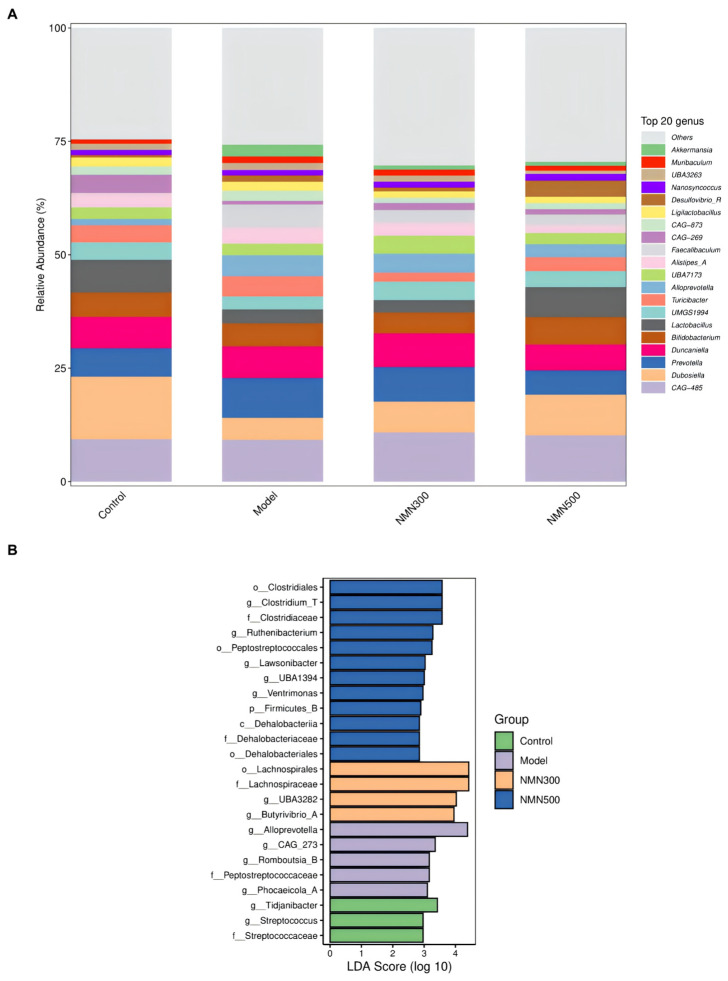
NMN remodels gut microbial composition and identifies key discriminatory taxa. (**A**) Relative abundance of the top 20 bacterial genera across the four experimental groups. Genera with relative abundance < 1% in all samples were grouped as “Others”. All bacterial names are presented in italics in accordance with standard taxonomic convention; (**B**) bar chart showing LDA effect value of marker species; *n* = 7.

The relative abundance of the top 20 bacterial genera across the four experimental groups is presented as a stacked bar plot in Figure 9A. In the control group, the gut microbiota was predominantly composed of beneficial and commensal genera, including *Ligilactobacillus*, *Lactobacillus*, *Bifidobacterium* and *Alistipes_A*, indicating a well-balanced microbial community structure. Following D-galactose administration, the model group exhibited a marked shift in composition, characterized by a visible expansion of polysaccharide-degrading and potentially pro-inflammatory taxa such as *Prevotella* and *Alloprevotella*, accompanied by a corresponding decline in several of the aforementioned beneficial commensals. Supplementation with NMN at 300 mg/kg resulted in an intermediate microbial profile that partially mitigated the D-galactose-induced dysbiosis, as evidenced by a modest restoration of certain beneficial genera, though the overall community structure remained more closely aligned with that of the model group. In the NMN 500 mg/kg group, the overall microbial community profile shifted markedly away from the dysbiotic pattern observed in the model group and toward a configuration that closely resembled that of the control group. Specifically, the pronounced expansion of *Prevotella* and *Alloprevotella* observed in the model group was substantially attenuated following high-dose NMN supplementation, with their relative abundances returning to levels comparable to the control group. Concurrently, the relative contribution of *Muribaculum* was maintained at an abundance similar to that observed in the control group, in contrast to its altered representation in the model group. Genera with a mean relative abundance below 1% across all groups were aggregated into the category “Others”. All bacterial names are presented in italics in accordance with standard taxonomic conventions.

LEfSe analysis revealed distinct taxonomic profiles across the four groups (Figure 9B). The control group was characterized by enrichment of *Streptococcus* and *Tidjianibacter*. In the model group, *Alloprevotella* and *Peptostreptococcales* were overrepresented. NMN at 300 mg/kg promoted the growth of *Lachnospirales*, *Lachnospiraceae*, and *Butyrivibrio_A*, whereas the 500 mg/kg dose enriched *Clostridiales*, *Clostridium_T*, and *Dehalobacteriota*. Collectively, these findings indicate that NMN induces dose-dependent remodeling of gut microbial composition and diversity, contributing to an improved intestinal microenvironment [39].

### 3.5. Association of Systemic Inflammation and Oxidative Stress with Gut Microbiota Functional Alterations

**Figure 10:**
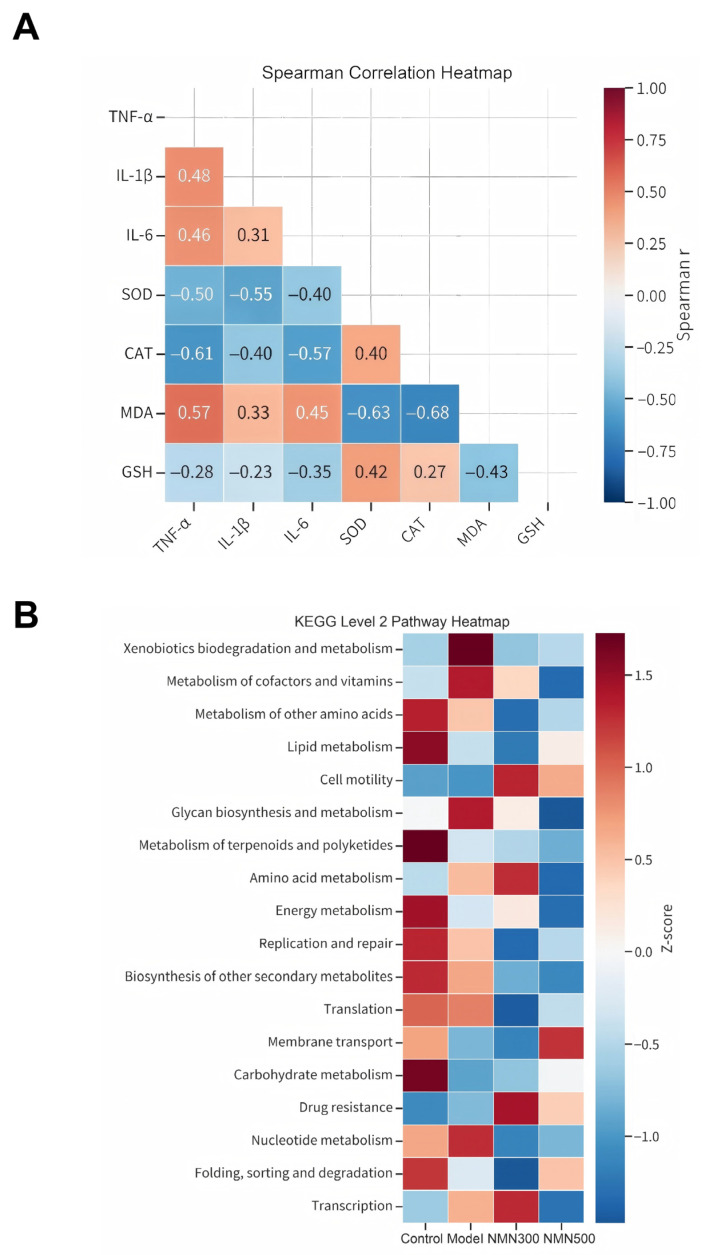
Analysis of correlations between inflammatory/oxidative stress markers and Spearman correlation heatmap (**A**), and functional prediction of gut microbiota using PICRUSt2 (**B**). (**A**) Spearman correlation heatmap among serum inflammatory cytokines and oxidative stress markers; (**B**) heatmap of KEGG Level 2 pathway abundance predicted by PICRUSt2 based on 16S rRNA sequencing data. The heatmap displays the relative abundance (represented as Z-score) of the top 18 metabolic and genetic information processing pathways across four groups: control, model, NMN300, and NMN500. Red indicates higher relative abundance (upregulation), while blue indicates lower relative abundance (downregulation). The color gradient represents the Z-score values ranging from −1.0 to 1.5; *n* = 7.

To explore the potential association between peripheral inflammatory/oxidative status and gut microbial function, we performed a Spearman correlation analysis between serum markers (inflammatory cytokines and antioxidant enzymes) and the relative abundance of KEGG Level 2 metabolic pathways (Figure 10).

The heatmap (Figure 10A) reveals distinct correlation patterns. Notably, pro-inflammatory cytokines TNF-α, IL-1β, and IL-6 showed strong positive correlations with pathways related to “Xenobiotics biodegradation and metabolism” and “Lipid metabolism”, while exhibiting negative correlations with “Carbohydrate metabolism” and “Energy metabolism”. Conversely, the antioxidant marker SOD was positively associated with energy-related pathways but negatively correlated with xenobiotic metabolism.

Furthermore, the functional prediction profile (Figure 10B) illustrates that the model group had a distinct microbial functional signature compared to the control group, characterized by an upregulation of metabolism-related pathways. NMN treatment appeared to attenuate these alterations, restoring the abundance of specific metabolic pathways (e.g., energy metabolism and carbohydrate metabolism) towards levels observed in the control group, suggesting that NMN may ameliorate cognitive impairment by modulating gut microbiota function and subsequently regulating systemic inflammation and oxidative stress.

### 3.6. NMN Upregulates the Nrf2/HO-1 Axis in a Mouse Model of Cognitive Impairment

**Figure 11:**
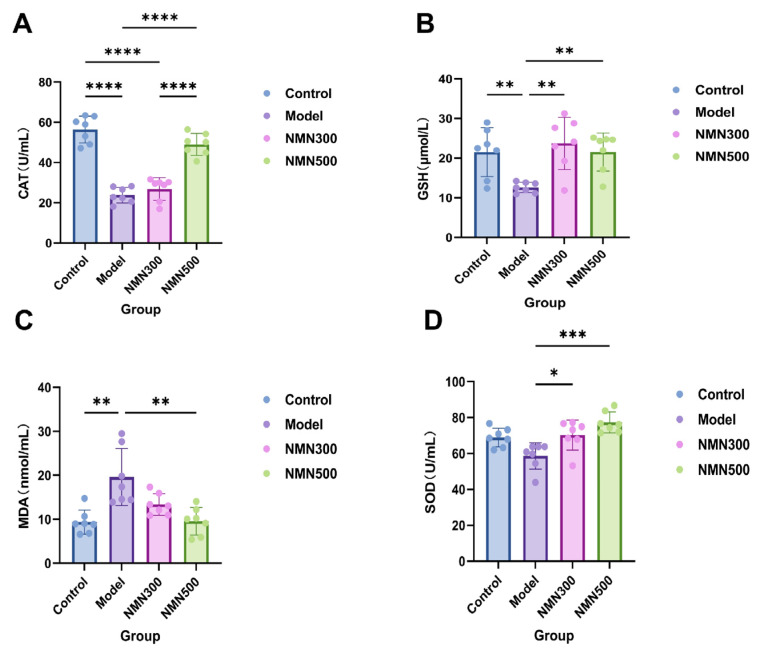
NMN can alleviate the oxidative damage in D-gal-induced aging mice. (**A**) Serum CAT levels measured by ELISA; (**B**) serum GSH levels measured by ELISA; (**C**) serum MDA levels measured by ELISA; (**D**) serum SOD activity measured by ELISA. NMN supplementation significantly increased CAT, GSH, and SOD levels and decreased MDA levels in D-galactose-induced aging mice. * *p* < 0.05, ** *p* < 0.01, *** *p* < 0.001, **** *p* < 0.0001; *n* = 7; data were analyzed by one-way ANOVA, followed by Tukey’s HSD post hoc test for multiple comparisons. The mean of the data is expressed with its standard deviation.

Oxidative stress (OS) is the harm to the body caused by excessive reactive oxygen species (ROS) and their derivatives, free radicals, as one of the reasons for aging and neurodegenerative diseases. During the metabolism of D-gal in vivo, it is oxidized by galactose oxidase to generate radicals such as hydrogen peroxide. The accumulation of ROS (Reactive Oxygen Species) destroys the cell’s redox homeostasis, causing lipid peroxidation and protein and DNA oxidation; it also damages cellular functions. To determine whether the antioxidant NMN can alleviate D-gal-induced oxidative damage in the body by measuring serum oxidative stress indicators. As shown in Figure 11, D-gal supplementation significantly decreased glutathione (GSH) levels and the activities of superoxide dismutase (SOD) and catalase (CAT) in the serum of aged dementia mice compared with the control group, thereby alleviating the oxidative stress state of aged dementia mice. Simultaneously, NMN showed a capability to reduce the levels of malondialdehyde (MDA) in the aged mouse model and improve oxidative damage; thus, it may exert a protective effect on brain tissue by scavenging free radicals.

**Figure 12:**
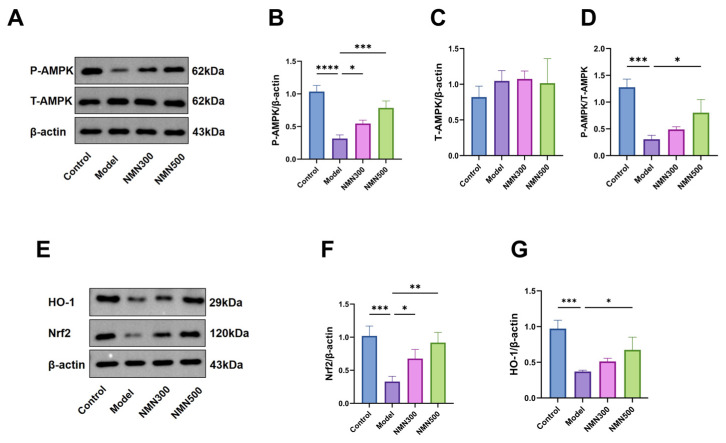
NMN activates the Nrf2/HO-1 pathway. (**A**) P-AMPK and T-AMPK expression levels in the brain were measured by Western blotting; (**B**) a semi-quantitative analysis of the P-AMPK gray value; (**C**) a semi-quantitative analysis of the T-AMPK gray value; (**D**) quantification of the P-AMPK/T-AMPK ratio; (**E**) HO-1 and Nrf2 expressions in the brain were detected by Western blotting; (**F**) a semi-quantitative analysis of the HO-1 gray value; (**G**) a semi-quantitative analysis of the Nrf2 gray value; * *p* < 0.05, ** *p* < 0.01, *** *p* < 0.001, **** *p* < 0.0001; *n* = 7; data were analyzed by one-way ANOVA, followed by Tukey’s HSD post hoc test for multiple comparisons. Data are expressed as means ± standard deviations.

As the central regulator of cellular energy homeostasis, AMP-activated protein kinase (AMPK) orchestrates oxidative stress responses and mitochondrial function. To determine whether NMN influences AMPK-mediated neuroprotection, we examined P-AMPK and total AMPK expression via Western blot (Figure 12A–D). D-galactose significantly suppressed P-AMPK and Nrf2 levels, whereas NMN supplementation reversed this suppression dose-dependently.

To investigate whether NMN correlates with Nrf2/HO-1 signaling, we performed Western blot analysis. D-galactose supplementation significantly reduced Nrf2 and HO-1 protein expression in the brain [40], whereas NMN elevated both factors in a dose-dependent manner (Figure 12E–G). This coordinated up-regulation suggests that NMN has a neuroprotective effect in the D-galactose-induced aging model, and activates the Nrf2/HO-1 pathway to some extent.

## 4. Discussion

Aging is an irreversibly multi-directional biological process in which the body’s regulatory capacity decreases with increasing age [41]. As the leading risk factor for cognitive impairment in the elderly, aging profoundly increases susceptibility to neurodegenerative changes, including cognitive decline, behavioral abnormalities, and impaired daily functioning, which ultimately lead to deteriorating mental health and social capacity [42]. The pathogenesis of age-related brain dysfunction is closely intertwined with fundamental biological processes of aging. While the etiology of cognitive aging is multifactorial—including genetic predisposition [43], protein aggregation [44], and cerebrovascular alterations [45]—emerging evidence highlights chronic inflammation [46] and oxidative stress [47] as key drivers that accelerate neurodegeneration in the aging brain. Given that these aging-related mechanisms are central to cognitive decline during aging, targeting them has become a promising therapeutic strategy. Among all the current research on age-related cognitive impairment, NMN appears to be one of the most promising. This study investigates whether NMN supplementation can attenuate D-galactose-induced hippocampal impairment, mitigate oxidative stress, and ameliorate gut microbiota dysbiosis, and explores the potential involvement of the Nrf2/HO-1 pathway in these effects.

More and more studies have shown that the interaction among gut microbiota, nervous system, immune system and endocrine system is essential for the normal development and function of homeostasis [48]. Regulatory interaction is called the microbiota-gut–brain axis, and it forms a two-way communication network of the enteric nervous system with the central nervous system to regulate the trajectory of health [49]. Maintaining the stability of this axis is essential for systemic homeostasis, influencing health span and the biological aging process [50]. Consequently, dysbiosis—characterized by structural and functional disturbances in the gut microbiota—can propagate pathogenic signals to the immune [51], endocrine [52], and nervous [53] systems, leading to various pathological outcomes, including impaired brain function [54]. As organisms age, the microbial ecosystem progressively deteriorates, increasing susceptibility to neurodegenerative and other age-related diseases [55]. Research on the role of the microbiota–gut–brain axis in cognitive deficits continues to evolve and has now been aimed at for preventive measures and therapies.

To elucidate the mechanisms by which changes in gut microbiota alter NMN-mediated neuroprotection, we performed comparative fecal 16S rRNA sequencing of D-gal-induced aged mice and healthy controls. Our analysis identified significant disease-associated shifts in gut microbial composition, which were partially reversed by NMN administration. Importantly, these microbial changes were significantly correlated with improvements in cognitive function and reductions in neuroinflammatory markers, suggesting a potential mechanistic association between microbiota remodeling and neuroprotection, although causality remains to be established.

To further explore the relationship between gut microbial functional potential and host physiological status, we performed Spearman correlation analysis integrating predicted KEGG pathways with serum markers of inflammation and oxidative stress (Figure 10). Pro-inflammatory cytokines (TNF-α, IL-1β, and IL-6) exhibited strong positive correlations with pathways related to xenobiotic biodegradation and lipid metabolism, whereas antioxidant markers (SOD and CAT) were positively associated with carbohydrate and energy metabolism pathways. In addition, PICRUSt2 functional prediction indicated that NMN supplementation partially restored the abundance of several metabolic pathways that were altered in the model group. While these functional inferences are based on 16S rRNA amplicon data rather than direct metagenomic or metabolomic measurements, the correlative patterns observed are consistent with the hypothesis that NMN-induced remodeling of the gut microbiota may contribute to the systemic attenuation of inflammation and oxidative stress via modulation of microbial metabolic output.

We hypothesized that NMN counteracts age-related cognitive decline by modulating redox signaling and activating the Nrf2/HO-1 antioxidant pathway [56]. In the D-galactose-induced aging model, the observed reduction in Nrf2/HO-1 expression was accompanied by microglial activation; NMN supplementation was associated with the restoration of Nrf2/HO-1 levels and attenuation of microglial activation.

An unexpected observation in our study was that NMN supplementation at 500 mg/kg reduced pro-inflammatory cytokine levels (IL-6, TNF-α, and IL-1β) slightly below those of the untreated control group. We offer several possible explanations for this phenomenon. First, NMN, as an NAD^+^ precursor, activates SIRT1 and the Nrf2/HO-1 pathway, which may suppress basal inflammatory tone beyond mere restoration of homeostasis. This anti-inflammatory effect has been reported in other studies where NAD^+^ boosting lowered baseline inflammatory markers even in healthy or aged animals. Second, the D-galactose-induced aging model may induce a mild chronic inflammatory state in what is considered the control group, due to systemic stress responses associated with daily injections and handling. Consequently, NMN supplementation could actively resolve this low-grade inflammation, resulting in cytokine levels that fall below those of the untreated controls. Third, the observed differences, while statistically significant in some pairwise comparisons, are biologically small and likely remain within the normal physiological range. Importantly, these reductions did not compromise normal immune function, as no adverse health effects were noted in NMN-treated animals. Further studies are needed to determine whether this “below-baseline” effect is beneficial or neutral in the context of aging. A more detailed mechanistic explanation of how D-galactose reduces antioxidant enzyme activity and how NMN alleviates this process is provided below. D-galactose disrupts the mitochondrial electron transport chain, leading to excessive generation of Reactive Oxygen Species (ROS), such as superoxide anions. These ROS not only cause direct oxidative damage to lipids, proteins, and DNA but also impair the nuclear translocation of nuclear factor erythroid 2-related factor 2 (Nrf2), thereby downregulating the expression and activity of antioxidant enzymes, including superoxide dismutase (SOD), glutathione (GSH), and catalase (CAT). This results in elevated malondialdehyde (MDA) levels and reduced antioxidant capacity. NMN Alleviates this process by acting as an NAD^+^ precursor. NMN elevates intracellular NAD^+^ levels, which activate the deacetylases SIRT1 and SIRT3. SIRT1 deacetylates Nrf2, promoting its nuclear translocation and subsequent transcriptional upregulation of antioxidant genes such as HO-1, SOD1, and CAT. SIRT3, which is localized in mitochondria, activates mitochondrial SOD2 (MnSOD) and isocitrate dehydrogenase 2 (IDH2), thereby enhancing mitochondrial intrinsic antioxidant capacity. Additionally, restoration of NAD^+^ levels improves mitochondrial respiratory function, reduces electron leakage, and decreases ROS production at the source. Thus, NMN simultaneously enhances cytosolic and mitochondrial antioxidant defenses, reversing the D-galactose-induced decline in antioxidant enzyme activity.

We acknowledge several pharmacological limitations in the present study. First, although the two NMN doses (300 and 500 mg/kg) were selected based on previous literature [37,38], we did not perform a systematic dose-response analysis, and the optimal dose for gut–brain axis modulation remains undefined. Second, we did not measure pharmacokinetic parameters or tissue exposure levels of NMN or its metabolite NAD^+^ in plasma or brain tissue. Therefore, we cannot determine whether the observed effects are directly mediated by NMN/NAD^+^ in the central nervous system or indirectly via peripheral mechanisms such as gut microbiota remodeling. These limitations weaken the pharmacological interpretation of our findings. Future studies incorporating a broader dose range, longitudinal sampling, and pharmacokinetic/pharmacodynamic measurements are necessary to establish a more robust basis for NMN’s protective effects in age-related cognitive decline.

In addition, the functional implications of the observed gut microbial shifts were inferred primarily from 16S rRNA taxonomic profiling and PICRUSt2 predictive functional analysis, rather than from direct quantification of microbial metabolites such as short-chain fatty acids. Although the enrichment of butyrate-producing taxa (e.g., *Butyrivibrio_A*, *Clostridium_T*, and *Lachnospiraceae*) and the predicted increase in butanoate metabolism provide compelling indirect support for enhanced butyrogenic capacity, direct measurement of SCFA concentrations in fecal or serum samples will be required in future studies to confirm the functional metabolic consequences of NMN-induced microbiota remodeling.

Furthermore, the present study employed a single-endpoint design, with all measurements collected after six weeks of concurrent NMN and D-galactose administration. This experimental framework does not allow for the determination of whether gut microbiota remodeling precedes or follows cognitive improvement. Longitudinal time-course sampling and fecal microbiota transplantation experiments will be necessary to disentangle the temporal dynamics and establish causality within the gut–brain axis.

In addition, although we performed Spearman correlation analysis to examine the interrelationships among serum inflammatory and oxidative stress markers (Figure 10A), direct statistical correlation between the relative abundances of specific gut microbial taxa (or predicted functional pathways) and host behavioral or biochemical parameters was not conducted in the present study. Such integrative correlation analyses would provide a more direct quantitative link between gut microbiota remodeling and the observed physiological improvements. We acknowledge this as a limitation and suggest that future studies incorporate comprehensive multi-omics integration approaches to fully delineate the microbiota–host interaction network.

Additionally, we did not directly measure ROS levels or assess mitochondrial respiratory function in this study. While the observed changes in antioxidant enzyme activities and NAD^+^ levels indirectly reflect oxidative status and mitochondrial health, direct quantification of ROS and functional assessment of mitochondria would provide more definitive evidence for the proposed mechanisms. This represents another limitation that should be addressed in future investigations.

**Figure 13:**
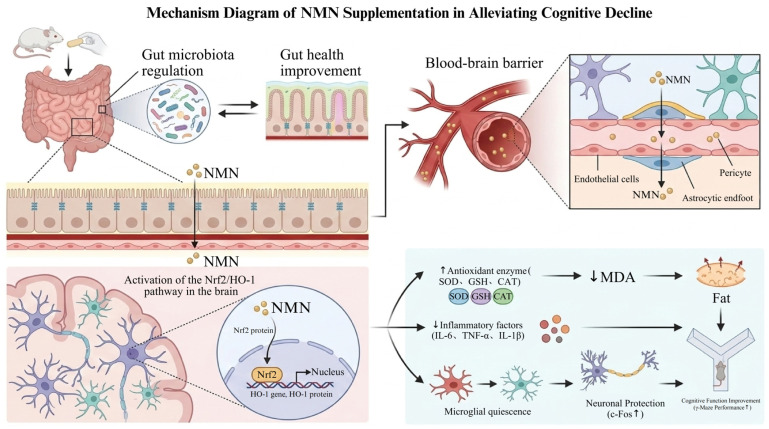
Mechanism diagram of NMN supplementation in alleviating cognitive decline. The schematic diagram was created using BioGDP.com (generic diagram platform).

The above results show that NMN reduces oxidative stress and neuroinflammation by activating Nrf2/HO-1, thereby mitigating gut dysbiosis. Thus, NMN appears to act through two convergent mechanisms—suppression of Nrf2/HO-1-mediated oxidative stress and attenuation of neuroinflammation—to restore gut–brain axis homeostasis. Histopathological and molecular analyses confirmed these neuroprotective effects, showing that NMN reduced microglial overactivation while improving cognitive function. Collectively, NMN preserves gut–brain axis integrity during aging (Figure 13).

Given the complex metabolic interplay between the gut microbiota and the central nervous system [57], our findings suggest that NMN-induced modulation of microbial composition may serve as an upstream event leading to subsequent neuroprotection in the aging brain. However, the specific probiotic and prebiotic strains that influence central nervous system signaling pathways, as well as the precise molecular mechanisms by which they regulate neural transmission, warrant further investigation.

Recently, many research findings have shown that NMN has neuroprotective effects on aging models, primarily attributing its effects to NAD^+^ replenishment, enhanced mitochondrial function, improved cerebrovascular integrity, and activation of sirtuin pathways. However, these investigations predominantly focused on vascular and metabolic aspects, with limited exploration of the gut microbiota as a potential mediator. In the present D-galactose-induced aging model, we not only corroborated the anti-inflammatory and antioxidant effects of NMN via the Nrf2/HO-1 pathway but also provided the first evidence, to our knowledge, that NMN profoundly reshapes the gut microbial ecosystem. Specifically, NMN supplementation enriched butyrate-producing genera such as *Butyrivibrio_A*, *Eubacterium_Q*, and *Clostridium_T*, which are known to exert anti-inflammatory effects and reinforce intestinal barrier function. This compositional shift closely paralleled the attenuation of neuroinflammation and cognitive improvement, suggesting that modulation of the gut microbiota may represent an additional, previously underappreciated mechanism underlying NMN’s anti-aging effects. Our findings extend the current knowledge by demonstrating that NMN not only preserves barrier integrity but also actively restructures the microbial community toward a more health-promoting profile. These observations position gut microbiota remodeling as a potentially integral component of NMN’s multimodal neuroprotective action.

A question that our data raises is whether changes in gut microbiota caused by NMN precede and cause neuroprotection, or whether it is merely an indirect consequence of the improvement of general health status. Although the present study did not directly measure blood–brain barrier (BBB) integrity, chronic D-galactose administration is known to impair BBB function, and our finding that NMN-enriched butyrate-producing taxa suggests an indirect protective effect on the BBB. Several pieces of evidence support the first possibility. First of all, the gut–brain axis is now more widely regarded as an important pathway for microbial metabolites to affect brain function. Butyrate and other short-chain fatty acids (SCFAs), which are produced by the taxa enriched in our NMN-treated groups, have been shown to enhance the blood–brain barrier, reduce microglial over-activation, promote the expression of tight junction proteins, and thus prevent peripheral inflammation from entering the brain. Secondly, new data show that SCFAs can directly activate the Nrf2 signal pathway in neurons and glial cells, providing a potential mechanism for the up-regulation of HO-1 mentioned in our study. Third, NMN may also directly affect intestinal epithelial cells through the increase of local NAD^+^ concentrations, improve mitochondrial function in these cells and reduce oxidative damage to the gut mucosa. A more suitable intestinal Environment can lower the likelihood that LPS and other pro-inflammatory factors will be absorbed into the bloodstream, reducing systemic inflammation and neuroinflammation. To sum up, modulation of the gut microbiota may be a type of upstream event that amplifies or even initiates the neuroprotective cascade triggered by NMN. To conclusively determine causality, future research can use fecal microbiota transplantation from NMN-treated donors to the recipient mice of germ-free or antibiotic supplementation. These experiments will determine whether the cognitive benefits of NMN can be transmitted through the microbiota entirely, and also confirm that the gut–brain axis must be considered as a therapeutic target for age-related cognitive impairment.

Although our 16S rRNA sequencing showed that there is a considerable NMN-induced reorganization of the gut microbiota, whether or not this change in microflora causes cognitive function changes still needs to be confirmed. New evidence shows that NMN may have anti-aging and neuroprotective effects, partially by regulating the gut microbiota-brain axis. NMN can improve the integrity of the intestinal barrier and reduce systemic inflammatory response to enhance brain function. In addition, it is known that D-galactose administration itself can damage the gut barrier function and microbial homeostasis, which also supports the idea that NMN’s cognitive benefits may be related to the improvement of gut ecology. In the future, to establish a causal relationship and prevent FMT from NMN-supplemented donors on germ-free or antibiotic-treated recipient mice. Such experiments can help determine whether the cognitive improvement benefits of NMN can be passed on when only the gut microbiota structure changes, thereby verifying that the gut–brain axis is a potential supplementation target.

This study provides novel insights into the mechanistic basis of NMN’s neuroprotective effects against age-related cognitive decline, to establish a cause-and-effect relationship of gut microbiota remodeling and cognitive improvement. Unlike previous studies that have reported associations between microbiota changes and brain aging, our work demonstrates that NMN directly correlates with the Nrf2/HO-1 pathway to alleviate oxidative stress and neuroinflammation, which in turn restores microbial homeostasis and preserves cognitive function during the aging process. This integrated mechanism underscores the protective effects of targeting the gut–brain axis as a strategy for mitigating age-related neurodegeneration.

Looking forward, the gut microbiota represents an attractive target for therapeutic intervention in age-related neurodegenerative diseases. Dietary modulation of the microbiota, such as through probiotic supplementation, may offer alternative or complementary strategies for mitigating age-related cognitive impairment, although long-term population-based studies are needed to validate these effects. Moreover, more mechanistic research needs to be conducted to determine if the improvement in memory function of D-gal-induced aging mice is related to changes in gut microbiota at the level of brain aging. A shortcoming of the present study is that only male mice were used, and thus, sex-related confounding factors in aging research cannot be excluded. Future research should therefore include both sexes to enhance the generalizability of the findings.

We also recognize that the D-galactose-induced aging model, while well-established for studying oxidative stress-driven cognitive decline, does not fully recapitulate the hallmark pathologies of human neurodegenerative diseases, such as amyloid-β plaques or tau neurofibrillary tangles. Consequently, the translatability of our findings to clinical Alzheimer’s disease or other dementias remains uncertain. Validation in disease-relevant transgenic models (e.g., APP/PS1 or 3 × Tg-AD mice) will be essential to assess the broader therapeutic potential of NMN in the context of specific neurodegenerative disorders.

Despite the observed upregulation of Nrf2 and HO-1 following NMN supplementation, which correlated with reduced oxidative stress and neuroinflammation, the present study does not provide direct mechanistic evidence that NMN exerts its neuroprotective effects exclusively through the Nrf2/HO-1 pathway. The use of pharmacological inhibitors (e.g., brusatol) or Nrf2-knockout mouse models would be necessary to establish causality. Should such interventions abolish or attenuate the antioxidant, anti-inflammatory, and cognitive benefits of NMN, it would strongly support the hypothesis that Nrf2/HO-1 signaling is a central mediator of NMN’s effects. Future studies should therefore incorporate loss-of-function approaches to validate the necessity of this pathway in NMN-mediated neuroprotection. It should be noted that only male mice were involved in the present study, and thus, our findings may have some limitations in their generalization. Sex differences have been reported in both aging processes and gut microbiota composition, as well as in responses to NAD^+^ precursor supplementation. For instance, estrogen has been shown to modulate Nrf2 signaling and microglial activity, potentially influencing neuroinflammatory outcomes. Therefore, the absence of female subjects may obscure sex-dependent effects of NMN on cognitive function and gut–brain axis regulation. Future investigations should include both sexes to assess potential dimorphisms in therapeutic response and to enhance the translational relevance of the findings.

## 5. Conclusions

In summary, the present study demonstrates that oral supplementation with nicotinamide mononucleotide during D-galactose challenge alleviates cognitive impairment in mice. These protective effects are associated with reduced oxidative stress and neuroinflammation, correlated with the upregulation of the Nrf2/HO-1 pathway, and accompanied by a remodeling of the gut microbiota toward a profile enriched in short-chain fatty acid-producing taxa. While the observed correlations between microbial shifts, predicted metabolic functions, and host physiological parameters support a potential role for the gut–brain axis in mediating NMN’s benefits, causality and the precise molecular mediators remain to be fully elucidated. Future studies incorporating direct metabolite quantification, longitudinal sampling designs, and disease-relevant models will be required to translate these preclinical findings into potential strategies for mitigating age-associated cognitive decline.

## Data Availability

The original contributions presented in the study are included in the article. Further inquiries can be directed to the corresponding authors.

## Abbreviations

The following abbreviations are used in this manuscript:

AD	Alzheimer’s Disease
AMPK	AMP-activated Protein Kinase
CAT	Catalase
CNS	Central Nervous System
DAPI	4′,6-diamidino-2-phenylindole
D-gal	D-galactose
DTT	Dithiothreitol
ECL	Enhanced Chemiluminescence
ELISA	Enzyme-Linked Immunosorbent Assay
EPM	Elevated Plus Maze
GSH	Glutathione
HO-1	Heme Oxygenase-1
HRP	Horseradish Peroxidase
IBA-1	Ionized Calcium-Binding Adapter Molecule 1
IF	Immunofluorescence
IHC	Immunohistochemistry
IL-1β	Interleukin-1β
IL-6	Interleukin-6
LDA	Linear Discriminant Analysis
LEfSe	Linear Discriminant Analysis Effect Size
MDA	Malondialdehyde
NAD^+^	Nicotinamide Adenine Dinucleotide
NeuN	Neuronal Nuclei
NMN	Nicotinamide Mononucleotide
Nrf2	Nuclear Factor Erythroid 2-Related Factor 2
OD	Optical Density
OE	Open Arm Entries
OS	Oxidative Stress
OT	Open Arm Time
OTU	Operational Taxonomic Unit
PBS	Phosphate-Buffered Saline
PBST	Phosphate-Buffered Saline with Tween-20
PCR	Polymerase Chain Reaction
PVDF	Polyvinylidene Fluoride
RIPA	Radioimmunoprecipitation Assay
ROS	Reactive Oxygen Species
rRNA	Ribosomal RNA
SDS-PAGE	Sodium Dodecyl Sulfate-Polyacrylamide Gel Electrophoresis
SOD	Superoxide Dismutase
TBST	Tris-Buffered Saline with Tween-20
TNF-α	Tumor Necrosis Factor-alpha
